# Excessive ADAM17 activation occurs in uremic patients and may contribute to their immunocompromised status

**DOI:** 10.1002/iid3.298

**Published:** 2020-03-17

**Authors:** Mitsuru Yashiro, Masaki Ohya, Toru Mima, Yuri Nakashima, Kazuki Kawakami, Tomohiro Sonou, Koichi Tatsuta, Yukiko Yamano, Shigeo Negi, Takashi Shigematsu

**Affiliations:** ^1^ Department of Nephrology Wakayama Medical University Wakayama Japan

**Keywords:** CKD, klotho, TACE

## Abstract

**Introduction:**

We previously reported that fibroblast growth factor 23 (FGF23)‐klotho signaling plays a role in B cell immunity. Despite high serum levels of FGF23, a decline in immunity is frequently observed in patients on hemodialysis (HD); thus, abnormalities in the FGF23‐klotho signaling pathway in immune cells may occur in these patients.

**Methods:**

We analyzed the number of klotho‐positive cells in peripheral blood mononuclear cells from 10 male and 6 female patients on HD and 5 healthy male subjects using flow cytometry. We analyzed the abundance of cleaved klotho protein in the murine B cell line, A20, and in the serum of HD patients and healthy subjects (HS) using flow cytometry and Western blotting. The serum level of A disintegrin and metalloprotease 17 (ADAM17) was measured in HD patients and HS using enzyme‐linked immunosorbent assay.

**Results:**

The number of klotho‐positive B cells was reduced in HD patients. Serum ADAM17 was responsible for the reduction in klotho, as a specific ADAM17 inhibitor reversed this change. The total serum levels of ADAM17 were similar in HD patients and HS; however, activated ADAM17 was increased in the serum of HD patients.

**Conclusions:**

We concluded that abnormal ADAM17 activation could contribute to the immunocompromised status in patients on HD, in line with the reported role of ADAM17 as an anti‐inflammatory and immunosuppressive factor.

## Graphical abstract

Klotho expression of lymphocyte was lower in the uremic patients (hemodialysis patient) than in healthy subjects, which was one of the causes for a decrease in a number of lymphocyte and immunocompromised status in uremic patients. It was one of the causes for the lower expression of klotho in lymphocyte of the patient that activated A disintegrin and metalloprotease 17 cleavaged klotho in lymphocyte.

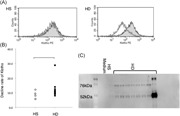

## 1 INTRODUCTION

Infection is the leading cause of death among patients undergoing hemodialysis (HD)^1^ and represents a critical issue in the clinical management of these patients. Chronic kidney disease (CKD) and a decline of renal function further increase the risk of death in HD patients.^2^, ^3^ Renal hyperparathyroidism and abnormal calcium phosphate metabolism are also among the possible causes of mortality^4^; however, this phenomenon has not been fully clarified in patients with CKD.

Fibroblast growth factor 23 (FGF23) plays a critical role in renal failure.^5^ Moreover, the serum level of FGF23 is relatively high in patients undergoing HD.^6^ We previously reported newly identified physiological roles of FGF23.^7^, ^8^ For example, FGF23‐klotho signaling in B cells plays an important role in the immune system. Although serum FGF23 levels were higher in HD patients compared with healthy subjects (HS), a decline in immunity was observed in these patients. Thus, we reasoned that abnormalities in FGF23‐klotho signaling could affect the immune system of HD patients, possibly increasing the risk of infection. The clarification of this aspect may contribute to both the prevention and treatment of infection in these patients.

In this study, we investigated the number of klotho‐positive cells in peripheral blood mononuclear cells (PBMCs) to verify the presence of FGF23‐klotho signaling abnormalities in the immune system of HD patients. Moreover, we sought to identify the molecular events underlying these abnormalities.

## 2 MATERIALS AND METHODS

### 2.1 Peripheral blood mononuclear cells

This study was conducted in accordance with the Declaration of Helsinki and was approved by the Institutional Review Board of Wakayama Medical University (Number 1785). Written informed consent was obtained from all participants.

Blood samples were obtained from 10 male and 6 female patients on HD and 5 male HS. PBMCs were isolated from peripheral blood using a Ficoll‐Paque gradient, according to the manufacturer's protocol (Amersham Pharmacia Biotech AB, Uppsala, Sweden). Aliquots of 10^5^ PBMCs were incubated with anti‐human Klotho/PE antibodies and anti‐CD3/FITC, anti‐CD19/FITC, or anti‐CD56/PE‐CF594 antibodies for 30 minutes on ice. Next, the samples were washed twice with Hank's balanced salt solution and analyzed with a FACSCalibur flow cytometer (BD Bioscience, CA).

### 2.2 Antibodies and reagents

The following antibodies were used in this study for the isotype control: anti‐CD3/FITC (Cat. no. 349201) for detection of T cells; anti‐CD4/FITC (Cat. no. 347413) for helper T cells; anti‐CD8/PE (Cat. no. 3473313) for cytotoxic T cells; anti‐CD19/FITC (Cat. no. 340409) for B cells; and anti‐CD56/PE‐CE594 (Cat. no. 561903) for NK cells. The mentioned antibodies were purchased from BD Bioscience. The anti‐Klotho antibody (Cat. no. KO603) was purchased from Trans Genic Inc (Kobe, Japan) and conjugated with PE using a Fluorescein Labeling Kit‐NH2 (Dojindo Molecular Technologies, Kumamoto, Japan).

### 2.3 Analysis of klotho in mouse B‐lymphoma cells exposed to the serum of HD patients

The mouse B‐lymphoma cell line, A20, was purchased from the Riken Cell Bank (Tsukuba, Ibaraki, Japan) and cultured in Roswell Park Memorial Institute 1680 medium supplemented with 10% fetal bovine serum (SH30079.03; HyClone, Logan, UT) at 37°C and 5% CO_2_. Briefly, A20 cells were cultured with 5% serum of HD patients for 24 hours at 37°C. Next, the cells were incubated with anti‐Klotho antibody using the above‐described method and analyzed on a FACSCalibur flow cytometer.

### 2.4 Western blot analysis of cleaved klotho

A20 cells were cultured with or without patient serum, as indicated above, and the media was collected. Next, 10 µL of culture medium was applied to each lane of polyacrylamide electrophoresis gels. After electrophoresis, proteins were transferred onto polyvinylidene difluoride (PVDF) membranes using the EzBlot reagent (AE‐1460; ATTO, Tokyo, Japan) and a semi‐dry blotting unit (WSE‐4110 Powered BLOT One; ATTO) according to the manufacturer's instructions. Membranes were blocked for 1 hour at room temperature in a commercial blocking reagent (PVDF Blocking Reagent for Can Get Signal; Toyobo, Osaka, Japan). The membranes were washed with Tris‐buffered saline (TBS; 50 mM Tris, 150 mM NaCl, pH 7.6) containing 0.05% Tween 20 (TBST) and then incubated with a rat anti‐mouse Klotho antibody (Trans Genic Inc [Kobe, Japan] KO603) diluted in immunoreaction enhancer solution (1:1000; Can Get Signal Solution 1; Toyobo), and kept overnight at 4°C. The membranes were washed with TBST and then incubated with horseradish peroxidase‐conjugated donkey anti‐rat immunoglobulin G (Abcam, Cambridge, UK) diluted in immunoreaction enhancer solution (1:5000; Can Get Signal Solution 2; Toyobo) for 1 hour at room temperature. Membranes were then washed in TBST and processed by an enhanced chemiluminescence protocol (ECL Prime Western Blotting Detection Reagent; GE Healthcare, Pittsburgh, PA). Antibody binding was detected using a WSE‐6100 LuminoGraph chemiluminescence imaging system (ATTO) and quantified by a CS Analyzer, version 1.0.2 (ATTO).

### 2.5 Detection of protease or metalloprotease in the serum of HD patients with cleaved klotho on A20 cells

A20 cells were cultured with 5% serum from HD patients in the absence or presence of 100 nM protease inhibitor (P1850; Sigma)/10 nM ethylenediaminetetraacetic acid (EDTA) or 10 nM TAPI‐0 (INH‐3850‐PI; Peptides international), to inactivate matrix metalloprotease or A disintegrin and metalloprotease 17 (ADAM17), respectively.

### 2.6 Quantification of ADAM17 by specific enzyme‐linked immunosorbent assay

The serum level of ADAM17 was measured in HD patients and HS using the Human TACE/ADAM17 Duo Set ELISA Development kit (DY930; R&D).

### 2.7 Analysis of both cleaved klotho and ADAM17 activity in the serum of HD patients

The activity of cleaved klotho was measured by the cleavage of the klotho index. Cleavage of klotho index = (log[peak intensity of A20 without patient serum] − log[peak intensity of A20 with patient serum]/log[peak intensity of A20 without patient serum]). In addition, the activity of ADAM17 was measured by the ADAM17 activity index. ADAM17 activity index = (log[peak intensity of klotho in A20 with patient serum] − log[peak intensity with both patient serum and ADAM17 inhibitor]/log[peak intensity of klotho in A20 with patient serum]).

### 2.8 Clinical data of patients included in this study

Clinical data were obtained from the 16 HD patients included in this study. To clarify the difference between the ADAM17 highly activated groups and ADAM17 poorly activated groups, sex, age, and laboratory parameters from the patients' notes were examined.

### 2.9 Statistical analysis

All values were expressed as the means ± SD, and statistical significance was determined using the Student *t* test. All statistical computations were performed using the Statistical Package for Social Sciences 21.0 for Mac (SPSS, Chicago, IL). *P* values less than .05 were considered statistically significant.

## 3 RESULTS

### 3.1 Flow cytometric analysis of PBMCs in HD patients

**Figure 1:**
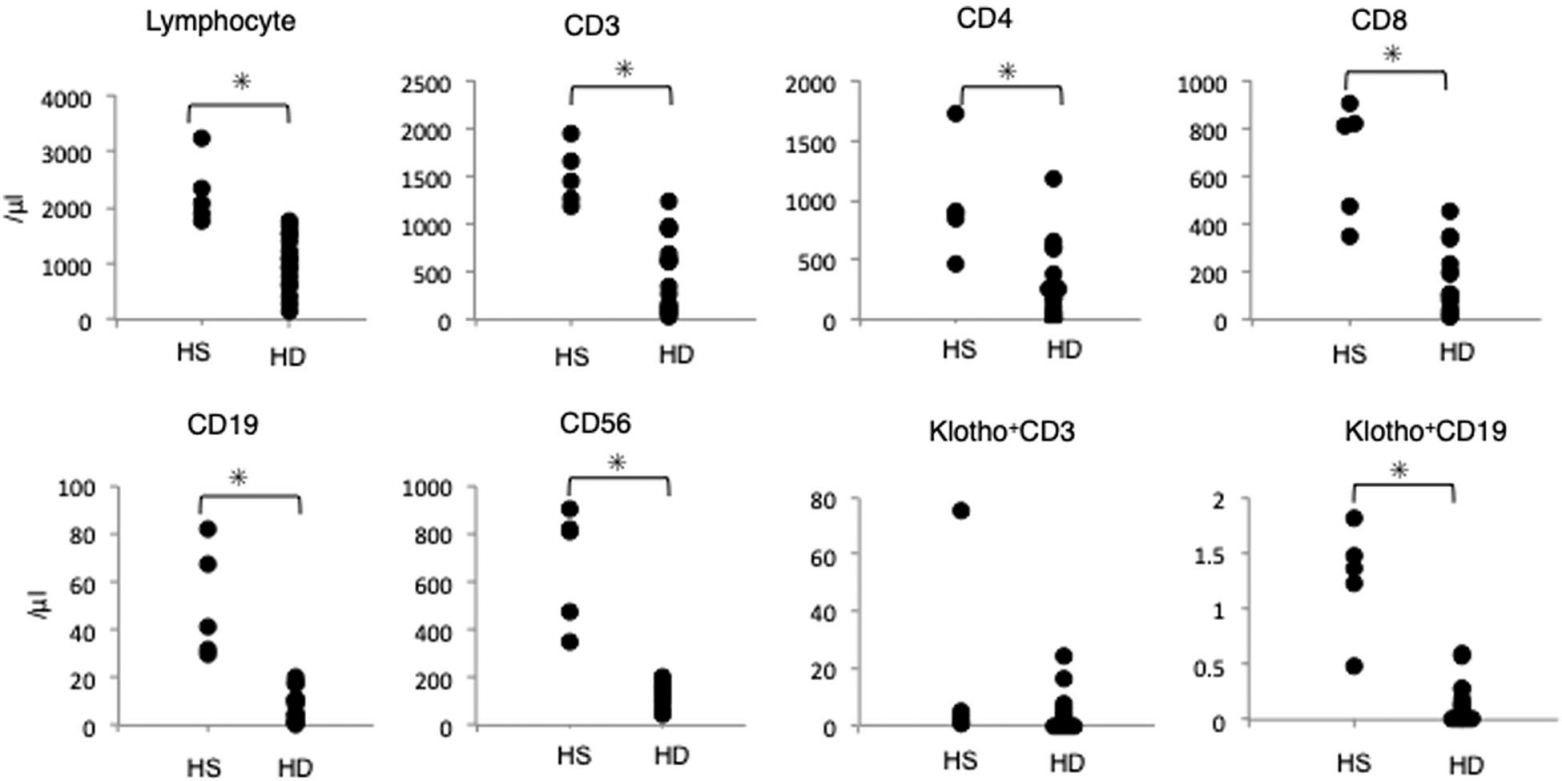
Peripheral blood mononuclear cells (PBMCs) and lymphocytes are decreased in HD patients. PBMCs were analyzed by flow cytometry. HD, hemodialysis; HS, healthy subjects. n = 5 to 16. *P < .05

The lymphocyte profile of 16 patients undergoing HD and 5 HS was analyzed by flow cytometry. The number of total lymphocytes was significantly lower in HD patients compared with HS (Figure 1). Moreover, the number of T, B, and NK cells, as well as the proportion of klotho‐positive B cells, was significantly reduced in HD patients compared with HS (Figure 1).

### 3.2 The patient serum reduced klotho expressions in the murine B cell line, A20

**Figure 2:**
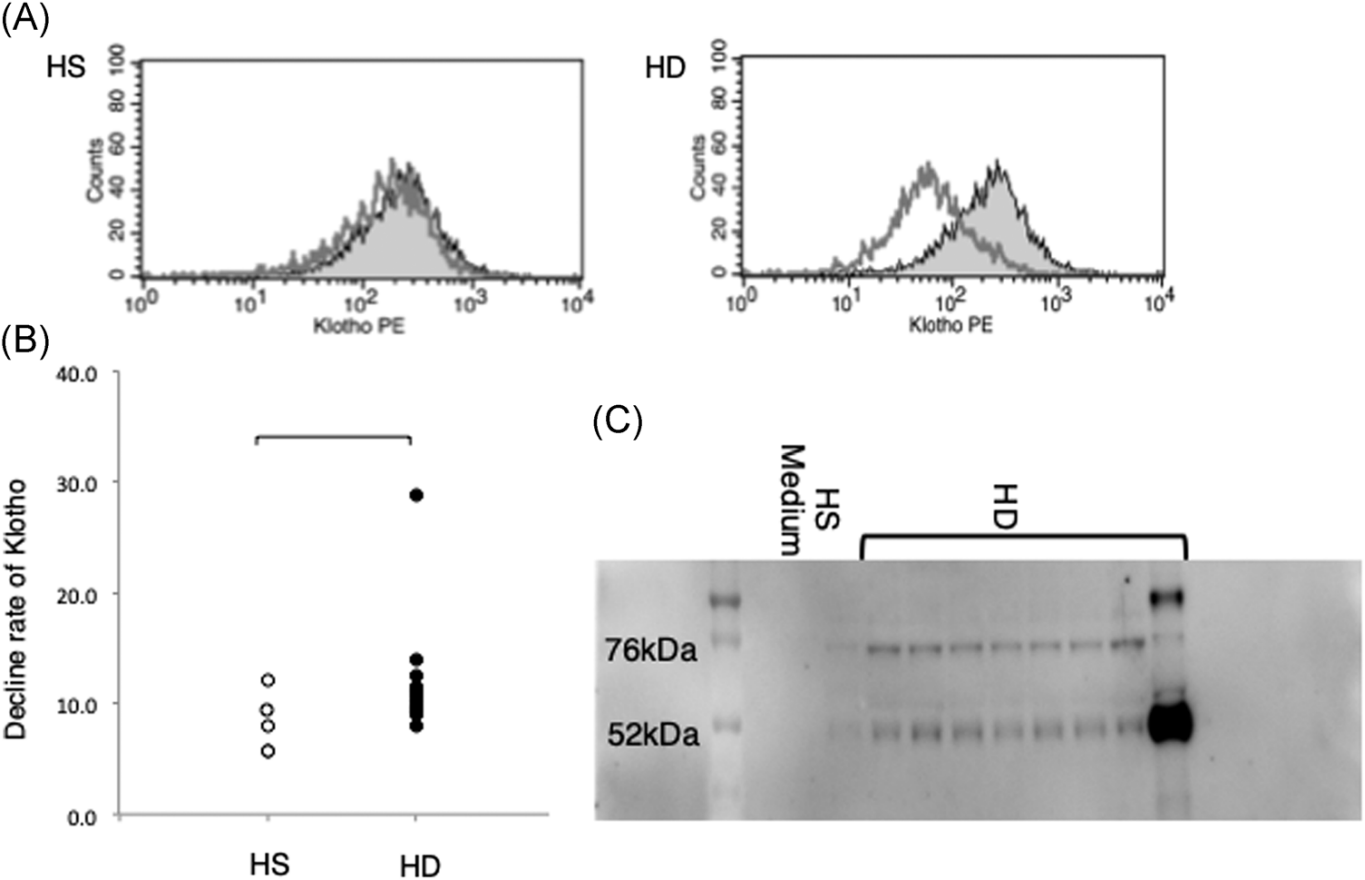
Decreased klotho in B cells resulting from klotho cleavage. A20 cells were cultured with or without 5% serum from patients on HD for 24 hours at 37°C. A, Klotho was measured by flow cytometry. Gray line area: cells cultured with serum; gray area: cells cultured without serum. B, Klotho decreased in A20 cells treated with HD patient serum. C, Cleaved klotho was detected in culture supernatants by Western blotting. HD, hemodialysis; HS, healthy subjects; medium, cultured medium

Of the 16 serum samples from HD patients, 8 were found to decrease the intensity of klotho expression, which correlated with the total quantity of klotho protein, in murine A20 cells (Figure 2). Conversely, the intensity of klotho in A20 cells remained unchanged following culture with serum from HS. Thus, the serum of a subset of HD patients caused klotho depletion in A20 cells, and this may be due to either transcriptional inactivation or proteolytic cleavage of klotho. Thus, Western blot was used to verify the presence of cleaved klotho in the media of A20 cells treated with klotho‐depleting patient sera. Indeed, klotho‐derived bands of 70 and 50 kDa were observed in these media, but not in the media of cells cultured with HS serum samples. Thus, proteolytic cleavage may account for the reduced klotho in B cells exposed to serum from HD patients.

### 3.3 ADAM17 is expressed in peripheral blood B cells from sera with klotho cleavage activity

To date, three types of klotho‐cleaving enzymes are known: beta‐secretase (BACE) and the metalloproteases, ADAM10 and ADAM17. The addition of BACE inhibitors to klotho‐cleaving sera from HD patients did not affect its ability to deplete klotho from A20 cells (Figure 3). Thus, BACE was not implicated in klotho cleavage. However, the presence of EDTA prevented klotho proteolyzes in these cells, indicating the involvement of a metalloprotease (Figure 3). Notably, tumor necrosis factor‐α (TNF‐α) processing inhibitor 0 (TAPI‐0), a specific inhibitor of ADAM17, also prevented klotho depletion in A20 cells. Therefore, we concluded that ADAM17 contained in patient sera could cleave klotho, thus, reducing its levels in lymphocytes.

Figure 3Cleavage of klotho from B cells by A disintegrin and metalloprotease 17 (ADAM17) contained in the serum of hemodialysis patients. A20 cells were cultured for 24 hours with 5% serum in the absence or presence of protease inhibitor, metalloprotease inhibitor, ethylenediaminetetraacetic acid (EDTA), or the ADAM17 inhibitor, tumor necrosis factor‐α processing inhibitor 0 (TAPI‐0). Klotho expression was measured by flow cytometry. A, Histogram of klotho expression. Blackline area: serum alone; dot line area: serum plus protease inhibitor; gray area: no serum. C, Histogram of klotho expression. Blackline area: serum alone; dot line area: serum plus EDTA; gray area: no serum. E, Histogram of klotho expression. Blackline area: serum alone; dot line area: serum plus TAPI‐0; gray area: no serum. B,D,F, Rate of klotho expression recovery in A20 cells. S, serum; S + E, serum plus EDTA; S + I, serum plus TAPI‐0; S + P, serum plus protease inhibitor

**Figure:**
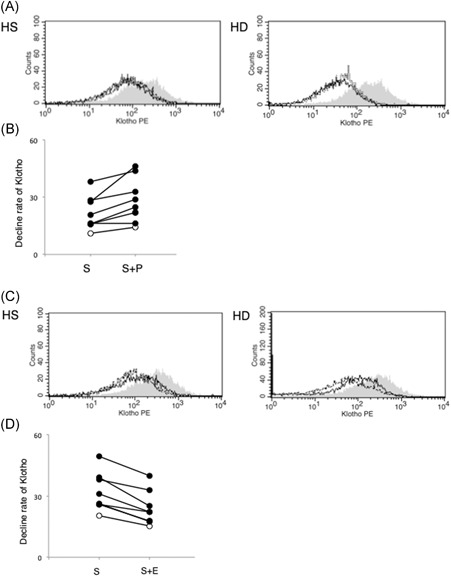

**Figure:**
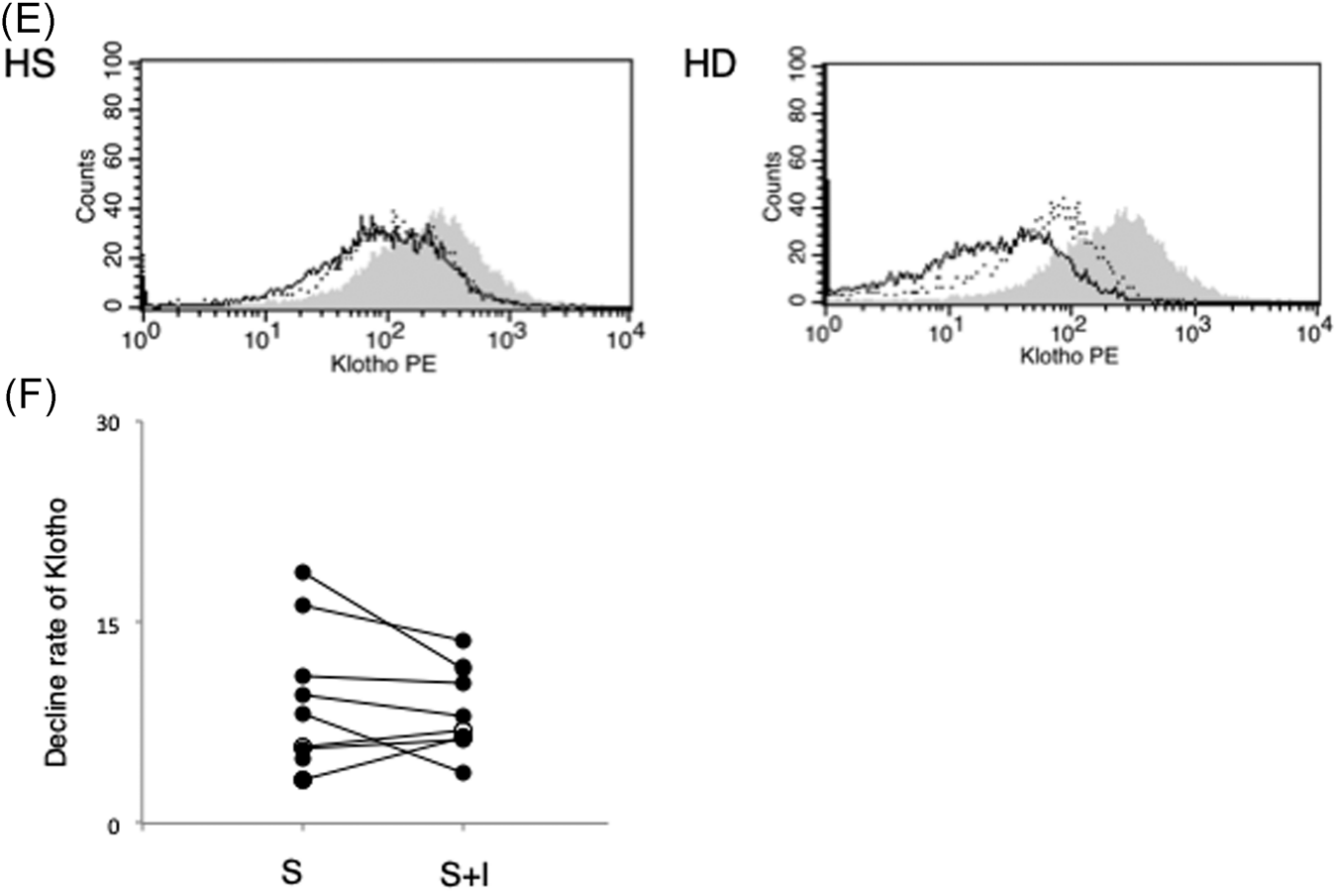

### 3.4 ADAM17 activity, but not its total level, was increased in the serum of hemodialyzed patients

**Figure 4:**
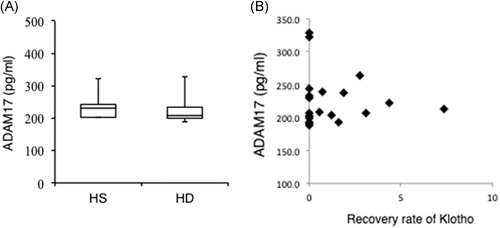
A disintegrin and metalloprotease 17 (ADAM17) content and klotho‐cleaving activity in serum. A, Serum levels of ADAM17 in both HS and HD patients were measured by enzyme‐linked immunosorbent assay. HD, hemodialysis; HS, healthy subjects. B, Dot plot showing total ADAM17 and recovery rate of klotho by ADAM17

The serum level of ADAM17 was measured by enzyme‐linked immunosorbent assay in both HS and HD patients. Although the total serum levels of ADAM17 were similar in the two groups, klotho cleavages by ADAM17 were more efficient in the sera of patients compared with HS (Figure 4). Thus, it was concluded that ADAM17 enzyme activity, rather than its total level, was altered in patients.

### 3.5 Clinical laboratory data from HD patients

To clarify the difference between the HD patients with highly active ADAM17 and ADAM17 with reduced activity, sex, age, and clinical laboratory data were investigated. HD patients with highly activated ADAM17 comprised more male patients with an overall younger age than patients with poorly activated ADAM17 (Table 1).

**Table 1:** Clinical laboratory data from hemodialysis patients with highly or poorly actived A disintegrin and metalloprotease 17

ADAM17 activity	High	Low
Sex (male:female)	7:1	3:5
Age, y	66.5 ± 11.45	72.9 ± 9.0
WBC, 10^2^/μL	80.3 ± 49.6	74.7 ± 42.0
Albumin, g/dL	3.06 ± 0.7	3.2 ± 0.6
CK, U/l	102.3 ± 81.5	85.3 ± 48.1
ALP, U/l	225.3 ± 60.6	220.8 ± 73.5 (n = 5)
Cre, mg/dL	9.3 ± 2.4	8.4 ± 3.3
UA, mg/dL	7.4 ± 1.4 (n = 7)	6.1 ± 1.2
BUN, mg/dL	60.0 ± 23.4	57.0 ± 27.3
Glucose, mg/dL	138.0 ± 35.8 (n = 6)	112.4 ± 34.8 (n = 5)
Ca, mg/dL	8.1 ± 0.8 (n = 7)	8.2 ± 1.0 (n = 6)
IP, mg/dL	6.2 ± 2.6 (n = 6)	4.8 ± 1.3 (n = 5)
Fe, μg/dL	71.5 ± 32.7 (n = 4)	86.8 ± 34.7 (n = 5)
CRP, mg/dL	1.0 ± 1.2	1.4 ± 2.0
Hemodialysis, y	10.9 ± 11.9 (n = 4)	4.0 ± 9.3 (n = 5)
Initiation of dialysis	4	3

## 4 DISCUSSION

To clarify the abnormalities in FGF23‐klotho signaling in the immune system of patients undergoing HD, PBMCs were analyzed by flow cytometry. PBMCs from HD patients exhibited reduced proportions of B, T, and NK cells, compared with healthy controls. Intriguingly, the number of klotho‐positive B cells was also reduced in HD patients, suggesting that the FGF23‐klotho signaling in lymphocytes in patients undergoing HD was intertwined.

The presence of factors in the serum of HD patients affecting the level of B cell‐derived klotho was verified by exposing mouse A20 cells to the serum of HD patients, followed by measurement of klotho by flow cytometry. Eight of the sixteen patient sera decreased the level of klotho, as well as the appearance of klotho cleavage products in the cell media. We concluded that proteolysis was responsible for the declining klotho levels in B cells. The enzymes with known klotho‐cleaving ability are the BACE protease, and the metalloproteases ADAM10 and ADAM17.^9^ The addition of protease inhibitors did not restore klotho levels in A20 cells exposed to HD serum. Conversely, the presence of EDTA prevented serum‐dependent klotho cleavage, suggesting the involvement of metalloproteases in this event. Notably, ADAM17‐specific inhibitors also prevented klotho cleavages, suggesting that ADAM17 in the serum of HD patients was responsible for klotho depletion from lymphocytes. ADAM17 activity was higher in the serum of HD patients; however, ADAM17 protein levels were similar in HS and HD patients. This suggested that abnormal ADAM17 activation, rather than a change in enzyme expression, was responsible for the decline in klotho‐positive lymphocytes and may contribute to the compromised immune response in HD patients.

**Figure 5:**
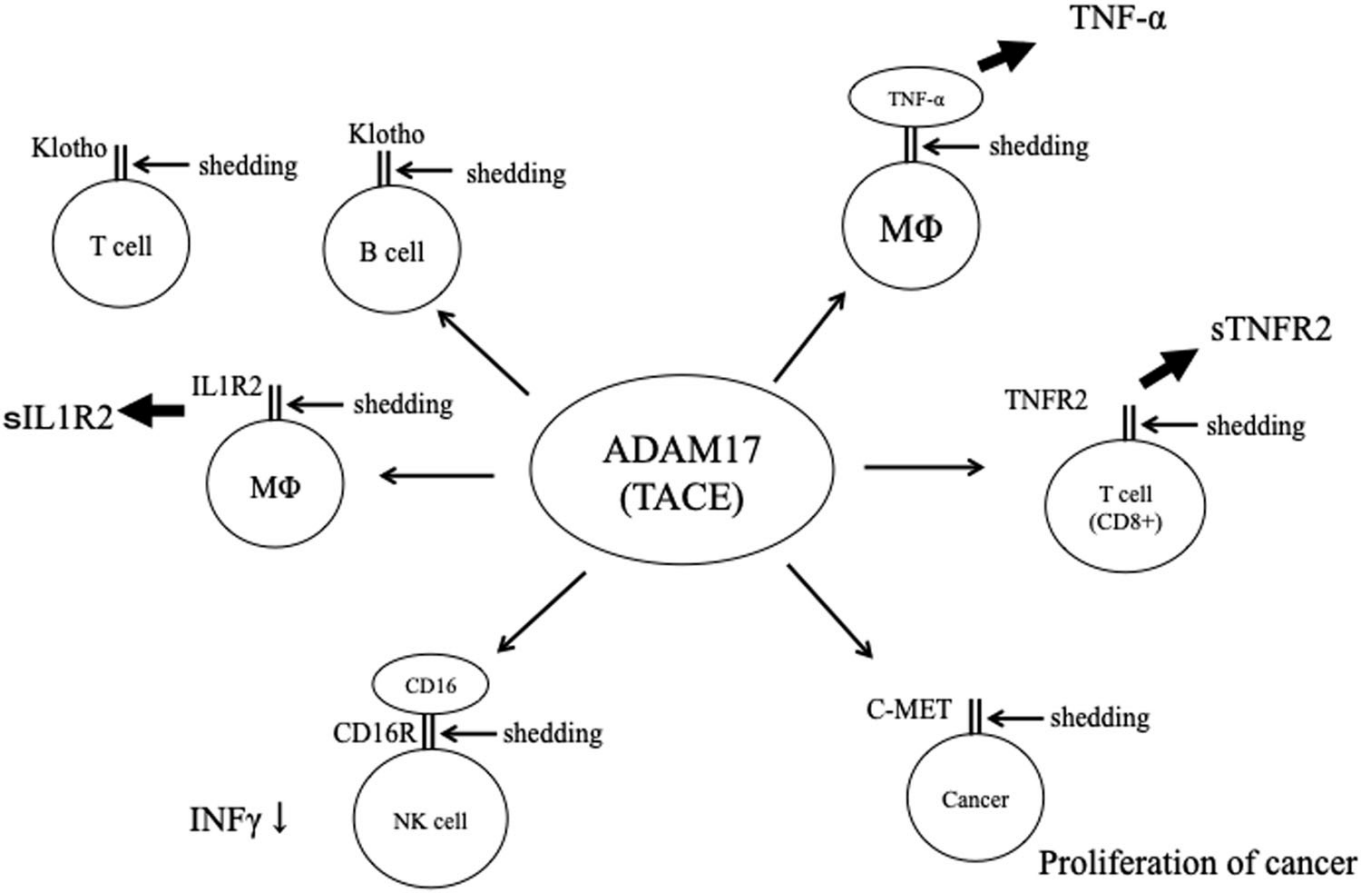
Summary of A disintegrin and metalloprotease 17 (ADAM17) actions as an immunoregulatory molecule and anti‐inflammatory agent. IL1R2, interleukin‐1 receptor 2; TACE, TNF‐α‐converting enzyme; TNF‐α, tumor necrosis factor‐α; TNFR2, TNF receptor 2

ADAM17 is involved in the shedding of TNF‐α precursors such as the TNF‐α‐converting enzyme (TACE).^10^ It was initially thought that inhibiting ADAM17 could suppress inflammation and TACE inhibitors were developed as anti‐inflammatory agents. However, unexpectedly, ADAM17 inhibition was also found to exacerbate inflammation.^11^ ADAM17 is involved in the cleavage of multiple targets including membrane‐bound ligands, cytokines, growth factors, receptors, and adhesion molecules.^12^ Currently, more than 80 ADAM17 substrates have been identified.^11^ For example, ADAM17 induces TNF receptor 2 shedding to neutralize TNF‐α and lymphotoxin‐α activity and releases interleukin‐1 (IL‐1) receptor 2 to inhibit IL‐1 activity.^11^ Moreover, CD16 cleavage by ADAM17 inhibits the activation of NK cells, and ADAM17‐induced proteolysis of CD40 causes defective B cell differentiation. Finally, the cleavage of CD34 by ADAM17 inhibits interferon‐γ production. These findings suggest that ADAM17 acts as a suppressor of inflammation^11^ (Figure 5). ADAM17 also disrupts the immune response to infection.

One factor that remains to be clarified is the excretion pathway of activated ADAM17. Some metalloprotease, such as matrix metalloprotease 3, are excreted in the urine^12^; therefore, it was thought that ADAM17 followed a similar mechanism. It is possible that delayed urinary excretion may induce excessively activated ADAM17 in HD patients. During HD, small weight molecules such as blood urea nitrogen, calcium, and phosphorus are removed by dialysis; however, larger weight molecules, such as metalloprotease, are not adequately removed by dialysis.^13^ This may also explain the increased activated form of ADAM17 in HD patients. Furthermore, circulating klotho from B cells was increased in uremic patients. However, the circulating klotho was primarily produced by renal tubular epithelial cells.^14^ Klotho‐positive renal tubular epithelial cells were reduced in HD patients concomitantly with the decreased renal function.^15^ Subsequently, circulating klotho from renal tubular epithelial cells is extremely reduced in uremic patients, resulting in the decreased total circulating klotho in the blood.

In the present study, we found that activated ADAM17 may deplete klotho from T and B cells, thereby suppressing immunity. The combined effect of ADAM17 on inflammation and immunity may contribute to the immune impairment that is observed in HD patients.

There are some limitations to this study. First, the molecular mechanism by which klotho affects immune response is unclear. Second, we did not clarify whether abnormal ADAM17 activation is directly involved in patient susceptibility to infection, and additional clinical research is needed to address this issue. Third, the cause of lymphocyte decline in the eight patients whose serum did not exhibit klotho‐cleaving activity is unknown. Finally, the mechanism of ADAM17 activation was not clarified by this study.

We found abnormalities in the FGF23‐klotho signaling in the immune system of patients undergoing HD. Activated ADAM17 was found to cleave klotho derived from lymphocytes in patients on HD. Therefore, abnormal serum levels of activated ADAM17 were thought to contribute to immune impairment in HD patients.

## CONFLICT OF INTERESTS

The authors declare that there are no conflict of interests.

## Data Availability

The data that support the findings of this study are available from the corresponding author upon reasonable request.

## References

[1] Masakane I , Taniguchi M , Nakai S , et al. Annual dialysis data report 2016, JSDT renal data registry. Ren Replace Ther. 2018;4:45.

[2] Collins AJ , Kasiske B , Herzog C , et al. Excerpts from the United States Renal Data System 2006 Annual Data Report. Am J Kidney Dis. 2017;49(A6‐7):S1‐296. 10.1053/j.ajkd.2006.11.019 17189040

[3] Sato T , Inoue T , Endo K , et al. End‐stage renal disease (ESRD) contributes to the increasing prevalence of herpes zoster. NDT Plus. 2009;2:263‐264. 25984009 10.1093/ndtplus/sfp024 PMC4421189

[4] Dalrymple LS , Katz R , Kestenbaum B , et al. The risk of infection‐related hospitalization with decreased kidney function. Am J Kidney Dis. 2012;59:356‐363. 21906862 10.1053/j.ajkd.2011.07.012 PMC3288732

[5] Moe S , Drüeke T , Cunningham J , et al. Definition, evaluation, and classification of renal osteodystrophy: a position statement from Kidney Disease: Improving Global Outcomes (KDIGO). Kidney Int. 2006;69:1945‐1953. 16641930 10.1038/sj.ki.5000414

[6] Imanishi Y , Inaba M , Nakatsuka K , et al. Increased levels of matrix metalloproteinase‐3 in sera from patients with active lupus nephritis. Clin Exp Rheumatol. 1998;16:409‐415. 9706420

[7] Nakashima Y , Mima T , Yashiro M , et al. Expression and localization of fibroblast growth factor (FGF) 23 and Klotho in the spleen: its physiological and functional implications. Growth Factors. 2016;34:196‐202. 28095739 10.1080/08977194.2016.1273222

[8] Yashiro M , Ohya M , Mima T , et al. FGF23 modulates the effects of erythropoietin on gene expression in renal epithelial cells. Int J Nephrol Renovasc Dis. 2018;11:125‐136. 29670389 10.2147/IJNRD.S158422 PMC5894721

[9] Bloch L , Sineshchekova O , Reichenbach D , Reiss K , Saftig P , Kuro‐o M , Kaether C . Klotho is a substrate for alpha‐, beta‐ and gamma‐secretase. FEBS Lett. 2009;583:3221‐3224. 19737556 10.1016/j.febslet.2009.09.009 PMC2757472

[10] Gooz M . ADAM‐17: the enzyme that does it all. Crit Rev Biochem Mol Biol. 2010;45:146‐169. 20184396 10.3109/10409231003628015 PMC2841225

[11] Zunke F , Rose‐John S . The shedding protease ADAM17: physiology and pathophysiology. Biochim Biophys Acta Mol Cell Res. 2017;1864:2059‐2070. 28705384 10.1016/j.bbamcr.2017.07.001

[12] Kotajima L . Increased levels of matrix metalloproteinase‐3 in sera from patients with active lupus nephritis. Clin Exp Rheumatol. 1998;16:409‐415. 9706420

[13] Azar AT . Dialyzer performance parameters In: Azar T, ed. Modelling and Control of Dialysis Systems. Berlin: Springer; 2013:379‐425.

[14] Shanahan CM . Mechanisms of vascular calcification in CKD‐evidence for premature ageing? Nat Rev Nephrol. 2013;9:661‐670. 24018414 10.1038/nrneph.2013.176

[15] Asai O , Nakatani K , Tanaka T , et al. Decreased renal α‐Klotho expression in early diabetic nephropathy in humans and mice and its possible role in urinary calcium excretion. Kidney Int. 2012;81:539‐547. 22217880 10.1038/ki.2011.423

